# Control of the MYC-eIF4E axis plus mTOR inhibitor treatment in small cell lung cancer

**DOI:** 10.1186/s12885-015-1202-4

**Published:** 2015-04-09

**Authors:** Masaru Matsumoto, Masahiro Seike, Rintaro Noro, Chie Soeno, Teppei Sugano, Susumu Takeuchi, Akihiko Miyanaga, Kazuhiro Kitamura, Kaoru Kubota, Akihiko Gemma

**Affiliations:** Department of Pulmonary Medicine and Oncology, Graduate School of Medicine, Nippon Medical School, 1-1-5, Sendagi, Bunkyo-ku, Tokyo 113-8603 Japan

**Keywords:** Small cell lung cancer, mTOR inhibitor, everolimus, MYC, eIF4E

## Abstract

**Background:**

Mammalian target of rapamycin (mTOR) inhibitors have anti-tumor effects against renal cell carcinoma, pancreatic neuroendocrine cancer and breast cancer. In this study, we analyzed the antitumor effects of mTOR inhibitors in small cell lung cancer (SCLC) cells and sought to clarify the mechanism of resistance to mTOR inhibitors.

**Methods:**

We analyzed the antitumor effects of three mTOR inhibitors including everolimus in 7 SCLC cell lines by MTS assay. Gene-chip analysis, receptor tyrosine kinases (RTK) array and Western blotting analysis were performed to identify molecules associated with resistance to everolimus.

**Results:**

Only SBC5 cells showed sensitivity to everolimus by MTS assay. We established two everolimus resistant-SBC5 cell lines (SBC5 R1 and SBC5 R10) by continuous exposure to increasing concentrations of everolimus stepwise. SPP1 and MYC were overexpressed in both SBC5 R1 and SBC5 R10 by gene-chip analysis. High expression levels of eukaryotic translation initiation factor 4E (eIF4E) were observed in 5 everolimus-resistant SCLC cells and SBC5 R10 cells by Western blotting. MYC siRNA reduced eIF4E phosphorylation in SBC5 cells, suggesting that MYC directly activates eIF4E by an mTOR-independent bypass pathway. Importantly, after reduction of MYC or eIF4E by siRNAs, the SBC5 parent and two SBC5-resistant cells displayed increased sensitivity to everolimus relative to the siRNA controls.

**Conclusion:**

These findings suggest that eIF4E has been shown to be an important factor in the resistance to everolimus in SCLC cells. Furthermore, a link between MYC and mTOR-independent eIF4E contribute to the resistance to everolimus in SCLC cells. Control of the MYC-eIF4E axis may be a novel therapeutic strategy for everolimus action in SCLC.

## Background

Lung cancer is a leading cause of death in Japan and the world [1]. Small cell lung cancer (SCLC), which is characterized as a neuroendocrine tumor, is one of the most aggressive cancers and is often diagnosed only in late stages. Metastases are frequently found on initial diagnosis of SCLC patients. Chemotherapy has a major role in treatment in advanced SCLC patients. Chemotherapy with cisplatin and etoposide or irinotecan has yielded the best outcomes in SCLC [2]; however, the major concern with these treatments is the short duration of response.

Molecularly-targeted therapies have been recently developed for non-small cell lung cancer (NSCLC) treatment. Oncogenic driver mutations including Epidermal growth factor receptor (EGFR) gene mutation and ALK translocation have been commonly found in NSCLC [3-5]. Recent randomized trials using gefitinib, erlotinib, afatinib, and crizotinib have demonstrated significant superiority of these molecularly-targeted drugs on progression-free survival compared with standard chemotherapies as key agents for advanced NSCLC with driver mutations [6-8]. However, there are oncogenic driver mutations found in SCLC, but they have not been successfully targeted. Therefore, molecularly-targeted agents developed for NSCLC are largely ineffective against SCLC. New targeted drugs are required for therapeutic strategies in SCLC.

Everolimus is a specific mammalian target of rapamycin (mTOR) inhibitor. It is approved for treatment of advanced renal cell carcinoma, pancreatic neuroendocrine cancer and breast cancer [9-11]. mTOR is expressed in approximately 50% of SCLC tumors, suggesting that the phosphatidylinositol 3-kinase (PI3K)/AKT/mTOR pathway is frequently activated in SCLC [12]. Everolimus has been evaluated as second-line therapy for SCLC in a phase II study [13]. One partial response was observed in a patient with sensitive relapse with an objective response rate of 3%. Disease control rate (DCR) at 6 weeks was 26%. Median survival was 6.7 months and median time to progression was 1.3 months. Baseline S6K expression was associated with DCR. Although the antitumor effect of everolimus was limited in that study, further evaluation of everolimus has been performed in combination regimens for SCLC patients designed to overcome drug resistance [14,15]. Therefore, identification of biomarkers predictive of sensitivity to everolimus could have a clinically significant impact on SCLC treatment strategies.

In this study, we analyzed the antitumor effects of three mTOR inhibitors including everolimus in SCLC cell lines and sought to clarify the mechanism of resistance to everolimus and thereby overcome such resistance. We ultimately found that MYC and eukaryotic translation initiation factor 4E (eIF4E) collaborate to offset the antitumor effect of everolimus and are promising therapeutic targets in SCLC.

## Methods

### Cell culture

We used 7 SCLC cell lines in this study. SBC3 and SBC5 were purchased from the Japanese Collection of Research Bioresources Cell Bank (Osaka, Japan). H69 and N231 were purchased from the American Type Culture Collection (Manassas, VA). MS1 and Lu139 were obtained from the Riken Cell Bank (Tsukuba, Japan). PC6 was purchased from Immuno-Biological Laboratories (Gunma, Japan). SBC5 and SBC3 were maintained in MEM-EAGLE medium (Sigma-Aldrich, Tokyo, Japan) with 10% fetal bovine serum (FBS; Gemini Bioproducts). The other SCLC cell lines were maintained in RPMI 1640 (GIBCO, Carlsbad, CA) with 10% FBS. These cell lines were obtained from 2008 to 2009, amplified and frozen, and one aliquot of each was thawed for this project. All cells were routinely screened for the absence of mycoplasma.

### Drugs and growth-inhibition assay

Everolimus, temsirolimus and rapamycin were purchased from Selleck Chemicals (S1120, S1044 and S1039) (Houston, TX). Growth inhibition was assessed by the MTS assay to examine the effect of everolimus, temsirolimus and rapamycin on SCLC cell lines as previously described [16]. Cell suspensions (5,000 cells/well) were seeded into 96-well plates and increasing concentrations of everolimus, temsirolimus and rapamycin (0, 0.001, 0.01. 0.1, 1.0, 10 and 100 μM) were added. After incubation at 37 °C for 72 h, MTS was added to each well and incubated at 37 °C for 2 h, after which absorbance was measured at a test wavelength of 490 nm using a microplate reader (Dynatech MR7000, Dynatech, Billinghurst, UK). The IC50 value was defined as the concentration of everolimus, temsirolimus or rapamycin needed for 50% reduction of growth and was calculated by SigmaPlot12 (HULINKS, Inc, Tokyo, Japan). Each experiment was performed independently three times. The corrected absorbance of each sample was calculated and compared with that of the untreated control.

### RNA isolation, cDNA array and RTKs phosphorylation antibody array

Total RNA was isolated from SCLC cell lines, as previously described [17,18]. High-density oligonucleotide array analysis was carried out using Affymetrix HG-U133A GeneChips (22,282 probe sets), as previously described [19]. We also performed human receptor tyrosine kinases (RTKs) phosphorylation antibody arrays, including 71 antibodies (RayBiotech, Inc. Norcross GA).

### Western blot analysis

Cells were lysed in buffer containing 50 mM Tris–HCl, pH 7.6, 150 mM NaCl, 0.1% sodium dodecyl sulfate, 1% Nonidet P-40, and 0.5% sodium deoxycholate. Western blot analysis was performed as previously described [18]. Antibodies detecting phosphorylated-AKT (p-AKT, Ser473), AKT, phosphorylated-EGFR (p-EGFR), EGFR, mTOR, phosphorylated-4E-BP1 (p-4E-BP1), 4E-BP1, c-MYC, phosphorylated-eIF4E were purchased from Cell Signaling Technology (Beverly, MA); Cat No. #9271, #9272, #2234, #2232, #2983, #2855, #9644, #9552, #9402, #9402. Antibody targeting β-actin was purchased from Sigma; Cat No. A5316.

### Oligonucleotide transfection

Small interfering RNAs (siRNAs) targeting MYC and eIF4E were purchased from Ambion (CA); c-MYC: Cat No. A) s9129, B) s9130; eIF4E: Cat No. A) s4576, B) s4577 and homologous negative controls were obtained from Invitrogen. siRNAs of MYC and eIF4E were transfected using Lipofectamine 2000 reagent 24 hours after seeding, according to the manufacturer’s instructions (Life Technologies, Carlsbad, CA). Transfections of siRNA complexes were added to cells at a final concentration of 50 nM.

### Fluorescence in situ hybridization (FISH)

Gene copy numbers (GCNs) and amplification of MYC gene were examined by FISH. Tissue sections were then hybridized with *MYC (8q24)* and *D8Z8 (8cen)* probes (LSI Medience Corporation, Chiba, Japan). Numbers of fluorescence signals were counted independently by two investigators using an Axio Vision microscope (Carl Zeiss, Oberkochen, Germany).

## Results

### Effects of mTOR Inhibitors on Small Cell Lung Cancer Cells and protein expressionn of AKT/mTOR pathway molecules

**Figure 1:**
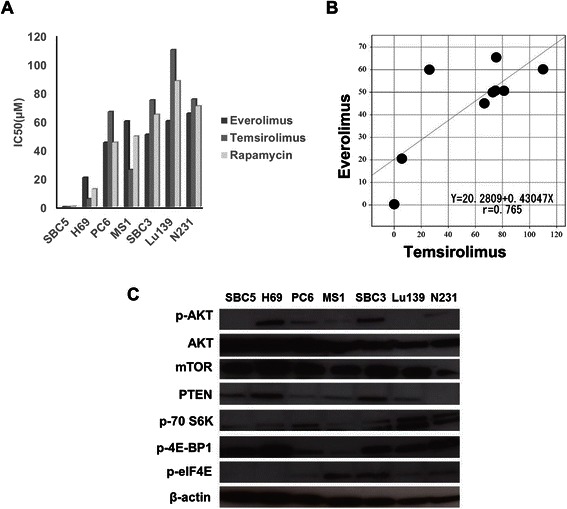
**Effects of mTOR inhibitors on SCLC cell lines and protein expression of PI3K/mTOR pathway molecules. (A)** IC50 values for 7 SCLC cell lines responding to mTOR inhibitor treatments by MTS assay. **(B)** Spearman correlation showed significant correlation between everolimus and temsirolimus. **(C)** Protein expression of PI3K/mTOR pathway molecules in 7 SCLC cells by Western blot analysis.

We examined the anti-tumor activities of three mTOR inhibitors including everolimus, temsirolimus and rapamycin against 7 SCLC cell lines by MTS assay (Figure 1A). Significant correlation of drug sensitivities was observed among the three mTOR inhibitors by Spearman correlation (Figure 1B). With reference to the Cmax of everolimus (70 nM), the 7 cell lines were classified as sensitive (IC50 ≤ 70 nM) or resistant (IC50 > 70 nM) to everolimus. Only SBC5 cells showed sensitivity to everolimus, whereas the other 6 cell lines showed resistance (Figure 1A). IC50 value of SBC5 cells for everolimus, temsirolimus and rapamycin were 4.9 nM, 9.3 nM, and 334 nM, respectively. We next evaluated protein expression levels of AKT/mTOR signal pathway molecules in the 7 SCLC cell lines by Western blot analysis (Figure 1C). Expression levels of p-AKT, AKT and mTOR did not differ remarkably among the 7 cell lines. Although expression of eukaryotic translation initiation factor 4E (eIF4E), a downstream component of the AKT/mTOR pathway, was not detected in SBC5 cells, its expression was remarkably increased in everolimus-resistant cells, with the exception of H69 cells. The IC_50_ value of H69 cells was lowest among 6 everolimus-resistant SCLC cells. However, high expression of p-AKT, the mTOR upstream molecule, was observed in H69 cells. Overexpression of p-AKT may affect the resistance to everolimus in H69 cells.

### Establishment of Everolimus-Resistant SBC5 Cells and Identification of Genes and RTK Associated with Resistance to Everolimus

**Figure 2:**
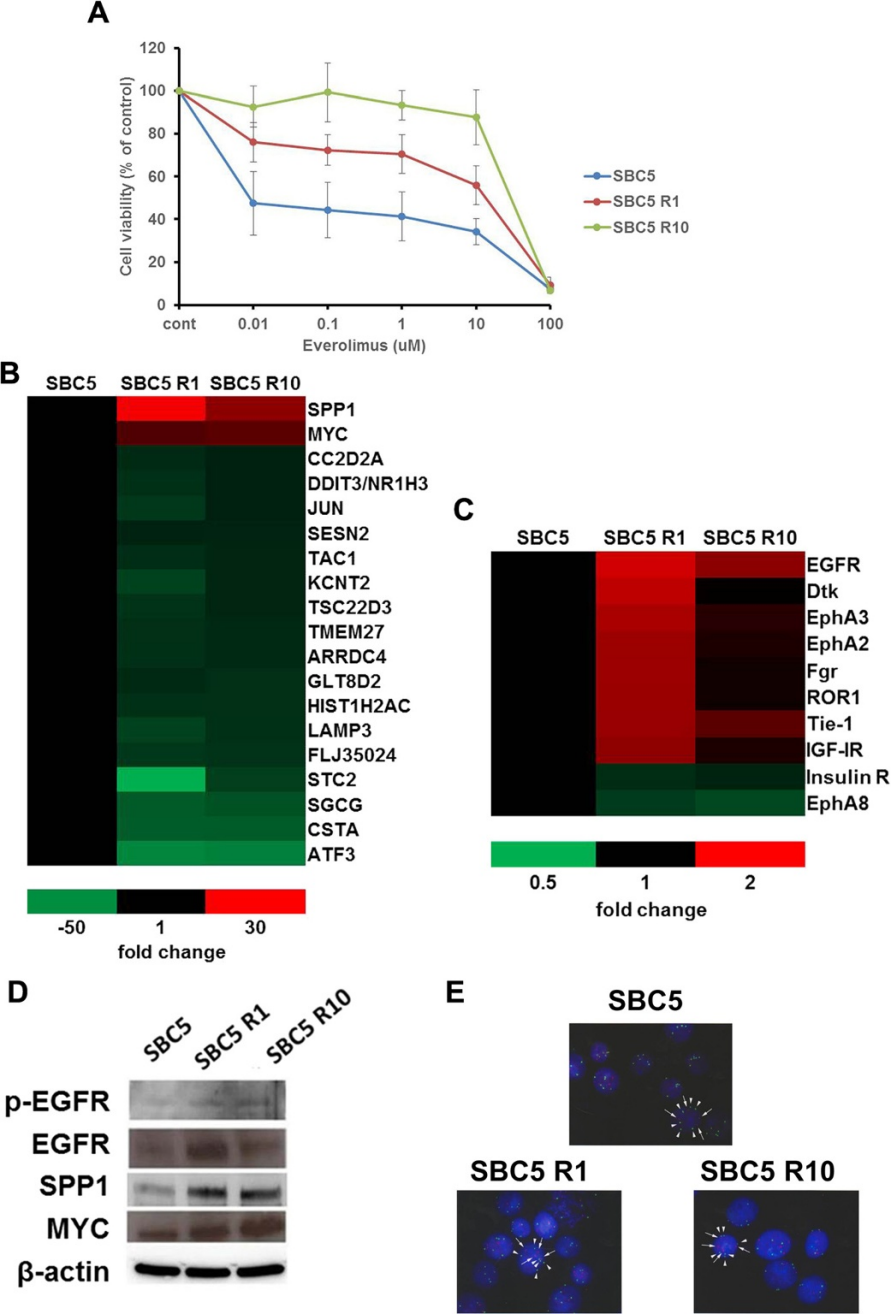
**Characteristics of SBC5 R1 and SBC5 R10 cells. (A)** MTS assays with everolimus in SBC5, SBC5 R1 and SBC5 R10 cells. Data are expressed as the mean ± SD from 3 independent experiments. **(B)** Differentially expressed genes between SBC5 parent and SBC5 resistant cells by Gene-chip analysis (Fold change >10, <-10). **(C)** RTK phosphorylation associated with everolimus resistance in SBC5 cells. Ten differentially expressed RTKs between SBC5 parent and SBC5 R1 cells by RTK array (Fold change >1.5, <0.8). Only p-EGFR was overexpressed in both SBC5 R1 and SBC5 R10 cells (1.82-fold and 1.55-fold, respectively). **(D)** Protein expressions of three candidate molecules in SCLC cells by Western blot analysis. p-EGFR, EGFR, MYC and SPP1 were elevated in the two everolimus-resistant SBC5 cells. **(E)** FISH analysis for MYC. Red signal indicates 8q24. Green signal indicates D8Z2 probe (8cen). No evidence of MYC overexpression was found.

To clarify the mechanism of resistance to everolimus, we sought to establish everolimus-resistant SBC5 cells by continuous exposure to increasing concentrations of everolimus stepwise. After two months, we established two SBC5-resistant cell lines which survived in either 1 μM (SBC5 R1), or 10 μM everolimus (SBC5 R10) (Figure 2A). We used these two SBC5 resistant-cell lines in further investigations. First, we performed gene expression profiles by Gene-Chip analysis to identify genes associated with resistance to everolimus. Expression of 19 genes differed significantly between parent SBC5 cells and SBC5 R1/SBC5 R10 cells (Fold change >10, <-10) (Figure 2B). Among the 19 genes, SPP1 and MYC were significantly overexpressed in both resistant cells. Second, we evaluated expression of phosphorylated RTK in SBC5 R1 and R10 cells versus parental SBC5 cells by RTK array (Figure 2C). Ten RTK were significantly changed in SBC5 R1 cells compared with parent SBC5 cells (Fold change >1.5, <0.8). Among the 10 RTK, only p-EGFR was also upregulated in SBC5 R10 cells (Fold change, 1.55). Based on these results, we focused on p-EGFR, SPP1 and MYC as everolimus-resistant candidate molecules. We next confirmed protein expression levels of p-EGFR, EGFR, SPP1 and MYC in SCLC cells by Western blot analysis (Figure 2D). p-EGFR and EGFR levels were increased in SBC5 R1 and SBC5 R10 cells compared to the parent cells. SPP1 and MYC were also elevated in SBC5 R1 and R10 cells with respect to the parent SBC5 cells. SPP1 as well as EGFR are known as upstream molecules of AKT/mTOR signaling and can activate downstream signals [20,21]. Overexpression of p-EGFR and SPP1 may be a result of negative-feedback effects of mTOR inhibition. In contrast, MYC can directly activate eIF4E, the most mTOR downstream molecule, via a bypass pathway [22]. We examined by FISH whether MYC amplification was observed as the mechanism of MYC overexpression in resistant cells. However, MYC gene amplification was not observed in either SBC5 resistant cell type (Figure 2E).

### MYC and eIF4E Contribute to Acquired Resistance to Everolimus

**Figure 3:**
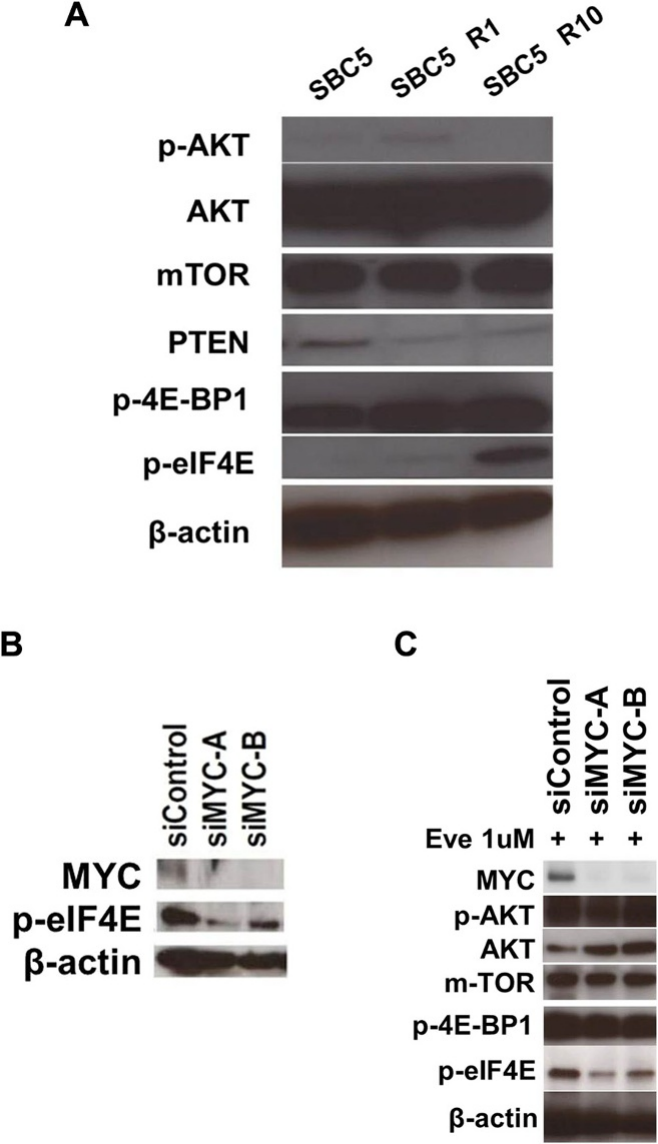
**MYC and eIF4E contribute to the acquired resistance to Everolimus. (A)** Protein expression of PI3K/mTOR pathway molecules in SCLC cells by Western blot analysis. p-eIF4E was significantly elevated in the two everolimus-resistant SBC5 cells. **(B)** Silencing of MYC by two siRNAs in SBC5 cells. p-eIF4E were decreased by si-MYC-A and si-MYC-B at 72 hours. **(C)** Silencing of MYC by two siRNAs in SBC5 R1 cells in the presence of everolimus 1uM. p-eIF4E protein levels were decreased by the treatments with the two si-MYCs at 48 hours. Eve: Everolimus.

We next examined protein expression levels of AKT/mTOR signal pathway molecules in both SBC5 resistant cells by Western blot analysis (Figure 3A). p-AKT, AKT and mTOR expression levels did not differ between parent SBC5 and SBC5 R1/R10 cells. In contrast, PTEN protein levels were decreased in both resistant cells. Suppression of PTEN resulting in AKT activation may be a result of negative-feedback effects of mTOR inhibition. Furthermore, eIF4E expression was elevated in SBC5 R10 cells over levels in parent SBC5 cells. Gene-chip analysis also revealed that resistance to everolimus resulted in increased eIF4E gene expression in SBC5 R1 and R10 cells by 2.86 and 2.86-fold, respectively (data not shown). A previous study demonstrated that eIF4E was directly regulated by MYC [22]. Expression levels of p-4E-BP1, an upstream direct inhibitor of eIF4E, were not changed in SBC5 R1 and R10 cells over levels in parent SBC5 cells (Figure 3A). These findings suggest that eIF4E may be directly regulated by a ‘bypassing’ pathway involving MYC in SBC5 resistant cells. Therefore, to further evaluate the effects of MYC and eIF4E on resistance to everolimus, MYC siRNAs were transfected into SBC5 and SBC5 R10 cells to examine whether MYC directly regulated eIF4E in the resistance to everolimus. Western blotting revealed that two si-MYCs reduced eIF4E phosphorylation in SBC5 cells (Figure 3B). AKT was overexpressed in SBC5 R1 cells treated with two si-MYCs in the presence of 1 μM everolimus (Figure 3C). Importantly, MYC was silenced in SBC5 R1 cells exposed to everolimus, showing decreased levels of p-eIF4E and no differences in levels of mTOR, or p-4E-BP1 (Figure 3C). These results suggest that eIF4E is directly activated by MYC in SBC5 and SBC5 resistant cells.

### Overcoming Resistance to Everolimus using MYC and eIF4E siRNAs

**Figure 4:**
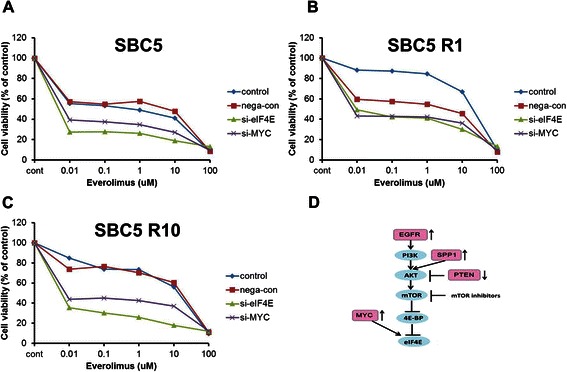
**MYC and eIF4E silencing increases the sensitivity to everolimus in SBC5 parent cells (SBC5) (A), SBC5 R1 cells (B), and SBC5 R10 cells (C) by MTS assay.** Data are expressed as the mean ± SD from 3 independent experiments. **(D)** Schematic of the results. eIF4F expression can be up-regulated by the PI3K/AKT/mTOR-dependent pathway by p-EGFR activation, PTEN suppression, and SPP1 overexpression. In contrast, MYC directly activates eIF4E expression by an mTOR-independent bypassing pathway.

Finally, we evaluated whether silencing of MYC and eIF4E could overcome the resistance to everolimus. SBC5 cells with reduced MYC or eIF4E following transfection with siRNAs displayed increased sensitivity to everolimus relative to siRNA controls (Figure 4A). Interestingly, SBC5 R1 and R10 cells treated with MYC or eIF4E siRNAs also reversed resistance to everolimus relative to siRNA controls (Figure 4B, C). These results suggest that MYC and eIF4E collaborate in drug resistance to everolimus, apparently bypassing the inhibitors in an mTOR-independent manner in SCLC cells (Figure 4D).

## Discussion

The commonly observed activated PI3K/AKT/mTOR pathway contributes to cancer development and maintenance in SCLC [12]. Activation of AKT through PI3K leads to increased mTOR activity through signaling by means of the TSC1/2 complex. mTOR phosphorylates S6K1 and 4E-BP1, and the most downstream effector, eIF4E. In this study, eIF4E levels were increased in 5 everolimus-resistant SCLC cell lines and in SBC5 R10 cells during everolimus therapy. eIF4E expression can be activated by mTOR-dependent and mTOR-independent pathways. Overexpression of p-EGFR and decreased PTEN can activate eIF4E through activation of the PI3K/AKT/mTOR pathway. Previous reports have demonstrated that SPP1 can also activate p-AKT and downstream molecules including eIF4E [20,21]. In contrast, we found that MYC directly activates eIF4E in SCLC cells by an mTOR-independent bypassing pathway.

MYC is one of the most frequently activated oncogenes and its expression correlates with poor prognosis in several human cancers [23]. MYC family DNA amplification has frequently been observed in SCLC patients and corresponding cell lines [24,25]. MYC amplification is also associated with poor survival in SCLC patients [24]. These findings suggest that MYC may be a driver gene and may constitute a possible avenue for therapeutic intervention in SCLC. MYC protein is a transcription factor that activates expression of many genes through binding on consensus sequences. MYC is considered to regulate the expression of 15% of all genes. However, despite the importance of MYC signaling in SCLC, MYC targets corresponding to drug resistance have not been fully clarified.

Our study showed that MYC directly activates eIF4E and contributes to drug resistance to everolimus in SCLC cells. Activation of both MYC and eIF4E have been found in human tumor cells [26,27]. A previous study reported that human mammary epithelial cells carrying the PIK3CA mutation showed acquired resistance to PI3K/mTOR inhibitor via the MYC-eIF4E axis [22]. The MYC-eIF4E axis contributes to resistance to PI3K/mTOR inhibitor via initiation of up-regulated cap-dependent translation [22]. In our study, reduction of MYC and eIF4E restored sensitivity to everolimus in SBC5 R1 and SBC5 R10 cells as well as in parent SBC5 cells. These findings suggest that assessment of eIF4E expression levels is useful for assessment of resistance to mTOR inhibitor, and control of the MYC-eIF4E axis is a promising therapeutic strategy for mTOR inhibition in SCLC.

Recently, aurora kinase inhibitors (AURKI) have been shown to be effective in SCLC cells with MYC amplification [28]. Direct links between MYC and aurora proteins have been reported [29,30]. In neuroblastoma, aurora kinase A expression is correlated with MYC amplification and protects the MYC protein from degradation [29,30]. Although the antitumor effect of everolimus was limited in SCLC, AURKI combined with everolimus may be useful for overcoming resistance to everolimus in SCLC with MYC activation and may constitute a new therapeutic strategy.

## Conclusion

In conclusion, eIF4E has been shown to be an important factor in the resistance to everolimus in SCLC cells. Furthermore, we found an important link between MYC and mTOR-independent eIF4E during resistance to everolimus in SCLC. Inhibition of the MYC-eIF4E axis may be a novel therapeutic strategy for overcoming the resistance to everolimus in SCLC with MYC overexpression. Further studies should be undertaken to clarify the mechanism of the connection between MYC and eIF4E and thereby establish a new therapeutic strategy for mTOR inhibition in SCLC.

## Abbreviations

mTOR: Mammalian target of rapamycin
SCLC: small cell lung cancer
RTK: receptor tyrosine kinases
EGFR: Epidermal growth factor receptor
eIF4E: eukaryotic translation initiation factor 4E
NSCLC: Non-small cell lung cancer
PI3K: Phosphatidylinositol 3-kinase
FISH: Fluorescence in situ hybridization, GCNs, Gene copy numbers
DCR: Disease control rate
